# P26 Challenging Scenario: Scleroderma/Sjogren overlap with dysautonomia

**DOI:** 10.1093/rap/rkac067.026

**Published:** 2022-09-28

**Authors:** Sujata Ganguly, Arumugam Moorthy

**Affiliations:** University Hospitals of Leicester NHS Trust, Leicester, United Kingdom; University Hospitals of Leicester NHS Trust, Leicester, United Kingdom

## Abstract

**Introduction/Background:**

Scleroderma Overlap conditions are well defined and often behave differently than their limited or diffuse counterparts. The treatment for such patients is usually tailored around the organ system involvement and severity. We present a challenging case of Scleroderma/Sjogren overlap with refractory Raynaud's phenomenon who posed treatment challenges due to severe autonomic dysfunction.

**Description/Method:**

76-year-old Caucasian lady, non-smoker, who presented in 1990’s with dry eyes, dry mouth and severe Raynaud's phenomenon with recurrent digital ulceration which became infected on multiple occasions. ANA was 1:400 with Extractable nuclear antigen Ro and La positive. Anti DsDNA was negative and C4 was low. Antiphospholipid antibodies and cryoglobulins are negative. She failed calcium channels blockers and phosphodiesterase 5 inhibitors and was intolerant to intravenous (IV) Iloprost which was tried on multiple occasions. IV latanoprost was tried as well. This treatment was complicated when she developed postural hypotension and multiple fall episodes during vasodilator use. This was attributed to autonomic dysfunction secondary to her Sjogren syndrome. She was initiated on Injection Fludrocortisone, however her hypotensive episodes continued. She received monthly intravenous immunoglobulins 20gm (over two days) infusion for a few months for autonomic dysfunction with some response. She eventually developed recurrent diarrhoea and abdominal pain and bloating which was attributed to small bowel overgrowth with rapid bowel transit on a gastric emptying scan. She had a blocked bile duct as well which was unblocked using Endoscopic Retrograde Cholangiopancreatography. Over the next 8 years, she continued struggling with refractory Raynaud’s, having failed IV regional guanethidine blocks and digital sympathectomy. Her postural hypotension decompensated on multiple occasions necessitating plasmapheresis which provided some benefit. In 2020, she was detected to have a PET avid slowly progressing left upper lobe lung lesion. She underwent left video assisted thoracoscopy surgery with upper lobectomy. Histology was suggestive of adenocarcinoma. In 2021, she had another fall episode due to an acute/subacute infarct of left pons. She continues having postural hypotension and requires regular plasmapheresis sessions to manage her symptoms. She is also presently on Bosentan for her raynaud’s phenomenon.

**Discussion/Results:**

This is a case of overlap of Sjogren syndrome with scleroderma features. Management of Refractory Raynaud’s as part of her scleroderma phenotype was challenging. This case gave us the opportunity to explore all the treatment options available for Raynaud’s phenomenon albeit with poor outcome. She also struggled with autonomic dysfunction leading to postural hypotension and gastrointestinal symptoms which can be seen in upto 50% of patients with Sjogren syndrome. Autonomic failure is often thought to be immune mediated in Sjogren syndrome. This makes way for the use of intravenous immunoglobulin and plasmapheresis to treat these conditions as was done in this case. Increased risk of lung carcinoma with scleroderma is well documented. Autonomic dysfunction can also present as paraneoplastic syndrome with lung carcinoma, however it is more common with small cell lung carcinoma and our patient was diagnosed with Adenocarcinoma. We would have to wait and see if the resection of the tumour has any demonstrable effect on her dysautonomia.

**Key learning points/Conclusion:**

Overlap connective tissue diseases are often difficult to manage in view of extensive disease heterogeneity. Clinicians need to be aware of challenges in managing Difficult Raynaud’s with Autonomic dysfunction. Treating physicians need to be aware of the high risk of lung malignancy and regular close monitoring is required. This case-based conference gives us the opportunity to discuss further management approaches for her.

